# Right in some respects: reasons as evidence

**DOI:** 10.1007/s11098-017-0954-x

**Published:** 2017-08-08

**Authors:** Daniel Whiting

**Affiliations:** Philosophy, Faculty of Humanities, University of Southampton, Southampton, SO17 1BJ UK

**Keywords:** Reasons, Evidence, Ought, Rightness

## Abstract

What is a normative reason for acting? In this paper, I introduce and defend a novel answer to this question. The starting-point is the view that reasons are right-makers. By exploring difficulties facing it, I arrive at an alternative, according to which reasons are evidence of respects in which it is right to perform an act, for example, that it keeps a promise. This is similar to the proposal that reasons for a person to act are evidence that she ought to do so; however, as I explain, it differs from that proposal in two significant ways. As a result, I argue, the evidence-based account of reasons I advance shares the advantages of its predecessor while avoiding many of the difficulties facing it.

## Introduction

That it is Elliot’s birthday is a reason for Stanley to buy him a present. That *Paris, Texas* is showing is a reason for Miyuki to go to the cinema. The reason in each case is a *normative* reason, a consideration which, to use Scanlon’s (1998: 17) well-worn phrase, ‘counts in favour’ of acting. But what is it for some consideration to favour, in the relevant sense, an action? What is a normative reason? In this paper, I present a novel answer to such questions.

One motivation for developing an account of reasons is that they seem to play a number of significant roles in our ethical lives, public and private. A person reflects on reasons when deciding what to do, acts or refrains from acting on the basis of reasons, appeals to reasons when explaining or defending her actions and those of others, and cites reasons when encouraging others to act or to refrain from doing so.^1^ It would be good to have an account of reasons which explains or reveals what makes them apt to play these roles, which in turn might illuminate these aspects of ethical life.

A further motivation for offering an account of reasons is that the notion of a reason stands in significant relations to others which figure prominently in ethical thought and talk, for example, rightness or value. A better understanding of the notion of a reason might facilitate a better understanding of these other notions, and vice versa.

Another concern which might lead one to advance an account of reasons is that the property of being a reason is in some way metaphysically or epistemologically puzzling. Without dismissing this concern, it will not frame or figure in the discussion to follow. The accounts of reasons I explore make ready appeal to other normative notions. It is an open question, which I will not tackle here, whether it is possible to offer an account of these further notions, or the phenomena they pick out, which addresses the relevant metaphysical or epistemological worries.

My starting-point is the view that reasons are right-makers.^2^ By exploring difficulties facing it, I arrive at an alternative, according to which reasons are evidence of respects in which it is right to act. This is similar to the proposal that reasons for a person to act are evidence that she ought to do so;^3^ however, as I explain, it differs from that proposal in two significant ways. As a result, I argue, the evidence-based account of reasons I advance shares the insights and explanatory virtues of its predecessor while avoiding the most serious difficulties facing it. More carefully, versions of some of those problems arise for my account but, I suggest, there are solutions to them which are unavailable to the proponent of its predecessor.

There are, of course, other theories of reasons in circulation.^4^ Since the aim is to advance a positive proposal, I consider critically alternatives only when doing so helps in motivating and assessing it. That is to say, the goal is not to defeat the competition, so to speak, but to introduce a new contender.

Before proceeding to the main discussion, I will address two preliminary matters. First, the views I consider here concern *objective* reasons, where an objective reason is a *fact*. Many hold that there are *subjective* reasons, where a subjective reason is an *apparent* fact, or what a person takes to be the case. What the relationship is between objective and subjective reasons is an interesting question, but not one I will try to answer here.^5^

Second, there is a familiar distinction between the reasons there *are* for a person to act and the reasons she *possesses* for acting, where a person possesses a reason for acting only if she is in a position to act for that reason.^6^ A common suggestion is that to possess a reason requires standing in an epistemic relation to the relevant consideration. What is that relation is another interesting question but, again, not one I will try to answer here.

## Reasons as right-makers

It is instructive to start with the idea that reasons are right-makers:

R = MR The fact that p is a reason for a person to φ if and only if it makes it right for her to φ.^7^

On this account, that *Paris, Texas* is showing is a reason for Miyuki to go the cinema just in case it makes it right for her to go.

Below I will raise some problems for R = MR. Before doing so, I will address some issues relating to it. First, one might advance R = MR as a truth that obtains in virtue of the *concept* of a reason. Alternatively, or in addition, one might advance it as a truth that obtains in virtue of the *property* of being a reason. Whether R = MR and the principles to follow are read as a conceptual or metaphysical claims makes little difference in what follows; where it does matter, I make a note of it.

Second, the notion of rightness figures in R = MR. Rightness is understood here as fact-relative. That is, whether it is right for a person to act depends on the facts (full stop), irrespective of a person’s epistemic relationship to the facts.^8^ Arguably, talk of rightness, so understood, is equivalent to talk of correctness or fittingness (cf. Chappell 2012; McHugh and Way 2016: 583). One might take this notion of rightness to be primitive, in the sense that one cannot explain it by appeal to other normative notions. If rightness is primitive in this way, there is a further question of whether it is possible to explain what rightness is in non-normative, perhaps naturalistic, terms. I take no stand on either issue here.

One might worry that R = MR not be fully adequate as an account of reasons unless the notion of rightness is basic or fundamental.^9^ However, it is not clear that an analysis is inadequate if it appeals to notions that in turn admit of analysis. The proposal that water is H_2_O seems a satisfactory and informative account of the nature of water, even if there is more to be said about the natures of hydrogen and oxygen. Be that as it may, even if R = MR falls short of a final analysis, it remains a non-trivial and substantive claim about the nature of reasons.

Third, which facts make it right to act? I take it that it is the job of first-order normative theory to answer that question. Perhaps there is a plurality of right-makers—that acting keeps a promise, that it benefits others, that it avoids harm, that it is fair, and so on. Perhaps there is one fundamental right-maker—that acting causes pleasure, say. The proponent of R = MR does not need to take a stand on these issues, and nor do I. Along the way, I will make some remarks about when it is right to act, but for illustrative purposes only.

Fourth, one might distinguish its being *morally* right to act, its being *prudentially* right to act, its being *aesthetically* right to act, and so on. On that basis, a proponent of R = MR might distinguish moral reasons, prudential reasons, aesthetic reasons, and so on (cf. Alvarez 2010: 15). Moral reasons are facts which make it morally right to act, prudential reason are facts which make it prudentially right to act, aesthetic reasons are facts which make it aesthetically right to act, and so on.

Finally, one might think that there are cases in which it is right for a person to φ and also right for her not to φ (cf. Alvarez 2010: 17). In such cases, it is right for a person to φ but not wrong for her not to φ. In view of this, a proponent of R = MR might distinguish between what one might call *justifying* reasons and *requiring* reasons.^10^ A justifying reason is a fact which makes it right to φ but not wrong not to φ, while a requiring reason is a fact which makes it right to φ and wrong not to φ. (So understood, requiring reasons are a subset of justifying reasons.) I am not defending this distinction—the point is that R = MR offers its proponent the resources for drawing it. For ease of presentation, I will for the most part set such matters aside in what follows.

## Problems for reasons as right-makers

An immediate problem with R = MR is that there can be a reason to act even though it is not right to do so. Suppose that it is not right for Miyuki to go to the pub. In that case, it cannot be right because she promised to do so. But that she promised is a reason for her to go. R = MR predicts otherwise.

The problem here is that being a reason is a contributory notion whereas being right (full stop) is an overall notion.^11^ In view of this, one might revise R = MR and suggest that reasons are facts which make it right *in some respect* for a person to act, where the notion of being right in a respect is contributory.^12^ More fully:

R = MRR The fact that p is a reason for a person to φ if and only if it makes it right in some respect for her to φ

A respect in which doing something has a certain property is a fact, one in virtue of which doing that thing possesses or exemplifies that property in some way or regard. For example, that cereal is high in fibre is a fact, one which makes eating cereal healthy in a way. So, it is a respect in which eating cereal is healthy, even if eating cereal is unhealthy in other respects or overall. Likewise, that the race is a marathon is a fact, one which makes her running it impressive in one regard. So, it is a respect in which her running the race is impressive, even if it is not impressive in every regard or overall.

To return to the example, that Miyuki promised to go the pub is a fact, one which makes it right for her to go to the pub in a way. So, it is a respect in which her going to the pub is right, even if it is wrong in another respect or overall. So, according to R = MRR, that Miyuki promised to go to the pub is a reason for her to go to the pub.^13^

R = MRR is a step in the right direction. But there is a further problem facing the proposal—it seems not to accord with our ordinary habits of treating certain considerations as reasons in thought, talk, and action. Many considerations which we treat as reasons are not respects in which it is right to act, or facts which make it right to act in some respect. Suppose, for example, that a fire alarm is sounding. Ananya might say or think that that is a reason for her to leave the building. When deciding what to do, she might take into account the fact that the alarm is sounding; it might figure as a premise in her practical reasoning. As a result of this deliberation, or via some other route, Ananya might leave the building for the reason that the alarm is sounding. When challenged, she might cite the fact that the alarm is sounding in justifying her behaviour. She might also appeal to that fact in trying to convince her colleagues to leave. In all these ways, Ananya treats the fact that the alarm is sounding as a reason for leaving. However, that fact is not plausibly a respect in which it is right for Ananya to leave the building or a fact which makes it right in some respect for her to do so; instead, it is *evidence* of some such respect, for instance, that by staying in the building she will suffer harm.^14^

To dwell on this, suppose that a first-order normative theorist arrives at a list of the fundamental respects in which it is right to act: that it avoids harm, that it keeps a promise, that it benefits others, that it is fair, and so on. That an alarm is sounding is not going to appear on this list.

Perhaps there are derivative right-makers—facts that stand in a suitable explanatory relationship to the fundamental right-makers. For example, that the food contains poison might be a derivative reason not to eat it *because* consuming poison causes pain, where the fact that some act causes pain is a fundamental reason not to perform it. However, the fact that the alarm is sounding does not stand in an explanatory relation to any of the items on the list; it does not explain why leaving the building keeps a promise, or benefits others, or avoids harm, or is fair, and so on. Rather, it stands in an *evidential* relation to certain items on the list; that the fire alarm is sounding indicates that leaving the building avoids harm, for example. To put the same point differently, it is not the case that leaving the building avoids harm (or keeps a promise, or benefits others, etc.) *because* the alarm is sounding; leaving the building does not avoid harm *in virtue of* the fact that the alarm is sounding. Rather, that the alarm is sounding is evidence that leaving the building avoids harm.

The point is not that, if reasons are facts which make it right in some respect to act, they cannot play the roles which reasons play in our ethical lives; rather, it is that many of the considerations which do play the relevant roles are not facts which make it right in some respect to act. R = MRR undergenerates reasons.^15^

Perhaps there is no requirement that an account of reasons respect all aspects of our ethical lives, or that it take our everyday habits of treating considerations as reasons at face value. However, if an account does accord with, or even vindicate, such things, that is a positive feature of it. In the next section, I will introduce such an account.

## Reasons as evidence

The above leads naturally to the view that a reason is evidence of a respect in which it is right to act. More fully:

R = ERR The fact that p is a reason for a person to φ if and only if:
  R is a respect in which it is right for her to φ;
  and the fact that p is evidence of R.

To illustrate, that an alarm is sounding is evidence that Ananya will avoid harm if she leaves. That she will avoid harm is a respect in which it is right for Ananya to leave. So, given R = ERR, that the alarm is sounding is a reason for her to leave. To return to an earlier example, that it is Stanley’s birthday is evidence of a respect in which it is right for Elliot to give him a present—that it will bring Stanley pleasure, say, or that it treats him fairly. So, given R = ERR, that it is Stanley’s birthday is a reason for Elliot to give him a present.

R = ERR is, of course, similar to R = MRR insofar as it appeals to the notion of rightness in a respect but there is an important difference. Whereas R = MRR involves the notion of making something the case, R = ERR involves the notion of being evidence that something is the case.

Like the proponent of R = MRR, a proponent of R = ERR might distinguish moral, prudential, aesthetic, etc., reasons for action by distinguishing respects in which it is morally, prudentially, aesthetically, etc., right to act, she might distinguish justifying and requiring reasons by distinguishing evidence of respects in which it is (merely) right to act from evidence of respects in which it is also wrong not to act, and she might distinguish objective and subjective reasons, or possessed and unpossessed reasons, by appeal to familiar distinctions between objective and subjective evidence, or between possessed and unpossessed evidence. Also, like the proponent of R = MRR, a proponent of R = ERR need not take a stand on what the respects are in which it is right to act, which is a question for first-order normative theory.

One might ask what it is for a fact to provide evidence for some proposition. I am not going to offer here an account of the evidential relation. Instead, I will rely on the intuitive idea that evidence to some degree makes it likely, renders it probable, indicates, etc., that a proposition is true. I do not think that anything will turn on the details of what evidence is.^16^

Note that evidence can be decisive. When there is decisive evidence that p, it follows that p. In this way, the fact that p is itself decisive evidence that p. So, R = ERR is consistent with the claim that a respect in which φing is right is itself a reason for φing, insofar as it is decisive evidence of a respect in which φing is right.^17^

To say that a reason for acting is evidence is not to say that the conclusion of practical reasoning is the belief or judgement the evidence supports. While R = ERR is silent on such matters, it is consistent with the view to hold that the conclusion of practical reasoning is the action or decision which the evidence, or the reasons it provides, supports. So, R = ERR does not turn practical reasoning into a species of theoretical reasoning.

R = ERR is similar to a recent and influential proposal which Thomson (2008) and, independently, Kearns and Star (2008, 2009) advance:^18^

R = EO The fact that p is a reason for a person to φ if and only if it is evidence that she ought to φ

To illustrate, that *Paris, Texas* is showing is evidence that Miyuki ought to go to the cinema. Hence, given R = EO, it is a reason for her to do so.

R = ERR is like R = EO insofar as it appeals to a normative notion and the notion of evidence. But R = ERR differs from R = EO in two important ways. The significance of these differences will become clear in what follows.

First, while R = EO appeals to an overall notion, specifically, that of what a person ought to do, R = ERR appeals to a contributory notion, specifically, that of a respect in which acting is right.

Second, according to R = EO, what makes something a reason is that it is evidence for a normative proposition, specifically, the proposition that a person ought to φ. According to R = ERR, in contrast, what makes something a reason is that it is evidence for a non-normative proposition. An example of evidence of a respect in which it is right to φ might be evidence that φing causes pleasure. That φing causes pleasure is a non-normative proposition. Of course, evidence of a respect in which it is right to φ can be evidence that φing is right (in some respect or overall). I do not deny this. The claim is only that the relevant consideration is not a reason in virtue of being evidence for such a normative proposition.^19^

## For reasons as evidence

Kearns and Star argue that R = EO enjoys several virtues. I will list some of those virtues and suggest that R = ERR enjoys them too.

First, Kearns and Star (2008: 38, 2009: §2.1) claim that R = EO points in the direction of a unified account of practical and epistemic reasons. This is true of R = ERR. Reasons of both kinds are evidence of respects in which it is right to do something. Practical reasons are evidence of respects in which it is right to act, while epistemic reasons are evidence of respects in which it is right to believe. On the plausible assumption that whether it is right to believe a proposition depends on whether it is true (cf. fn14 above), we get the welcome result that reasons for believing a proposition are evidence of truth. It is clear how R = ERR might generalise further to reasons for other attitudes (fear, admiration, etc.).

Second, Kearns and Star (2008: 39–41, 2009: §2.3) suggest that R = EO explains the role reasons play in deliberation. R = ERR also offers such an explanation. When Miyuki is deliberating as to whether to go the cinema, she considers reasons for or against going. This makes sense if, in doing so, she is considering evidence of respects in which it is right or wrong to go the cinema.

Third, Kearns and Star (2009: §2.4) claim that R = EO explains why we appeal to reasons in trying rationally to persuade a person to act. Again, R = ERR offers such an explanation. When attempting to convince Miyuki to go the cinema, I present her with reasons for going. This makes sense if, in doing so, I present her with evidence of respects in which it is right that she goes.

Fourth, R = EO allows ‘many of the mundane considerations crucial to ordinary ethical thinking’ to count as reasons (Star 2015: 37). R = ERR does the same. More generally, as the last two points illustrate, it does justice to the role that reasons play in our ethical lives, to the various ways in which we treat considerations as reasons. As discussed above, R = MRR falls short in this regard.

Fifth, a familiar thought is that reasons have weights. That *Paris, Texas* is showing is a reason of some weight for Miyuki to go the cinema, while that she promised to go to the pub is a weightier reason for her not go to the cinema, and to go to the pub instead. R = EO lends itself to a straightforward account of the weights of reasons. According to Kearns and Star (2008: 44–45, 2009: §2.6), the weight of a reason for a person to φ is its weight as evidence that she ought to φ.

R = ERR also lends itself to a plausible account of the weights of reasons, although this calls for more comment. First, recall the earlier point that, in some cases, a respect in which it is right to φ is also a respect in which it is wrong not to φ. Second, note that an act can be more or less wrong. For example, killing a person is more wrong than breaking a promise. In view of this, an attractive suggestion is that the weight of a reason is a function of its weight as evidence and the degree of wrongness. For illustrative purposes, suppose that a reason’s weight is the product of the other factors. If that p is weak evidence (0.2) of a respect in which not φing is very wrong (0.9), while that q is moderate evidence (0.5) of a respect in which φing is a bit wrong (0.2) the account predicts that that p is a weightier reason to φ than that q (0.18 > 0.1).

Of course, this is a crude sketch.^20^ It is beyond the scope of this paper to defend a view about what exactly the relevant function is; indeed, one might want to leave that open as a matter for dispute among proponents of R = ERR. The important point for present purposes is that R = ERR provides the basis for a promising account of the weights of reasons.

## Objections to R = EO

I have suggested that R = ERR shares the positive features of R = EO. R = EO also faces a number of objections that, individually or cumulatively, present a serious challenge to the view. Given the similarity between R = ERR and R = EO, one might wonder if my account faces versions of those objections. In this section, I will explore some difficulties facing R = EO and show that my account of reasons avoids them from the outset.

### Counterevidential reasons

There can be a reason for a person to φ which is not evidence that she ought to φ but evidence *against* this proposition (see Brunero 2009; Brunero Forthcoming; McKeever and Ridge 2012). To adapt Brunero’s example, suppose that a group of friends are deciding which film to watch. One of the friends, Juan, will enjoy *Transformers*. This is a reason for the group to pick that film. However, whenever Juan enjoys a film, everyone else hates it. So, that Juan will enjoy the film is not evidence that the group ought to pick *Transformers*; on the contrary, it is evidence that it ought not to do so, hence, that it is not the case that it ought to do so. Cases like this appear to be counterexamples to R = EO.

This problem does not arise for R = ERR. That Juan will enjoy *Transformers* is evidence of a respect in which it is right for the group to pick the film, say, that it will bring Juan pleasure. So, given R = ERR, it is a reason for the group to pick *Transformers*. Of course, that Juan will enjoy the film is also evidence of a respect in which it is wrong to pick the film, namely, that it will bring the other members of the group pain. So, it is also a reason not to pick the film. These are the right verdicts. One and the same consideration can be a reason to φ and a reason not to φ. Compare: that it is sunny is a reason to go outside, insofar as it is pleasant and helps the body produce vitamin D, and a reason not to go outside, insofar as it exposes skin and eyes to harmful ultraviolet rays.

R = ERR avoids the counterexample because the normative notion it involves is contributory, rather than overall.

### Weightlessness

Gregory objects to R = EO on the following grounds:

Conclusively defeated evidence has no normative force, whereas conclusively defeated reasons do have normative force. So reasons cannot simply be evidence of what one ought to do. (2016: 2303)

To appreciate this, consider a version of an earlier example. That *Paris, Texas* is showing is a reason for Miyuki to go the cinema. However, the evidence that Miyuki ought not to go the cinema is conclusive. In that case, the evidence that she ought to go the cinema is just misleading; Miyuki need accord it no weight in any further deliberation. From R = EO it seems to follow that she need accord the fact that *Paris, Texas* is showing no weight as a reason for going to the cinema in any further deliberation. But that is wrong. In Gregory’s words, that *Paris, Texas* is showing is not a mere ‘red herring’; it really does count in favour of going to the cinema. If Miyuki does what she ought to do and avoids the cinema, she might regret not going for the reason that *Paris, Texas* is showing.

If this objection undermines R = EO, does it also undermine R = ERR? No. Although, in the above case, that *Paris, Texas* is showing bears no weight as evidence that Miyuki ought to go to the cinema, it continues to bear weight as evidence of a respect in which it is right that she goes, say, that it will bring her pleasure. So, given R = ERR, it is a reason for Miyuki to go to the cinema, even when the evidence that she ought not to go is conclusive.

Of course, if there is conclusive evidence against the proposition that going to the cinema will be pleasurable for Miyuki, the fact that *Paris, Texas* is showing is misleading as evidence of a respect in which it is right for her to go. Hence, assuming it is not evidence of any other respect in which it is right that Miyuki goes, R = ERR predicts that it bears no weight as a reason for going. But that seems the right result; in that case the consideration really is a red herring.

Once again, R = ERR avoids the problem at hand because the normative notion it involves is a contributory, rather than overall, notion.

### Misleading ethical evidence

Cases involving misleading normative evidence—that is, misleading evidence about the normative facts or the normative significance of the non-normative facts—create difficulties for R = EO. Consider an anti-Rossian view, according to which there is a prima facie duty of infidelity. On this view, that a person promises to do something is always a reason for her not to do it. The anti-Rossian view is, I take it, false but suppose that there is evidence for it—a distinguished ethicist presents an argument for the view, say, or one’s peers behave in ways that suggest it is true.

Against the background of the evidence for the anti-Rossian view, the fact that Miyuki promised to meet Nishi at the pub is evidence that she ought not to go. So, according to R = EO, it is a reason for her not to go. But this is the wrong verdict—after all, the anti-Rossian view is false. R = EO seems to rule out the possibility that the evidence in this case is misleading.^21^

R = ERR avoids this problem. According to it, that Miyuki promised to meet Nishi at the pub is a reason for her not to go only if it is evidence of a respect in which it is right for her not to go, which, in the above case, it is not. If anything, that Miyuki promised to meet Nishi at the pub is evidence of a respect in which it is right that she goes, namely, that she promised to do so. Hence, given R = ERR, it is a reason for her to go, which seems the right verdict.

R = ERR avoids the problem since, according to it, what makes some consideration a reason for acting is not that it provides evidence for a normative proposition, as R = EO maintains, but that it provides evidence for a non-normative proposition which, if true, makes acting right in some way.^22^

### Reason implies can

Consider the principle that *reason implies can* (RIC). According to RIC, if there is a reason for a person to do something, she can do that thing. R = EO seems to clash with RIC (McKeever and Ridge 2012: §1.3). Suppose that Nishi will be at the pub. Given that Miyuki promised to meet Nishi, this is evidence that she ought to go to the pub. However, as it happens, Miyuki is unable to go. R = EO predicts that, in this case, that Nishi will be at the pub is a reason for Miyuki to do something she cannot do.

One option, which Kearns and Stars take (2009: §3.23), is to reject RIC. Insofar as the principle is independently plausible, this is a cost.^23^ Another option is to try to show that, despite appearances, R = EO is indeed consistent with RIC. Rather than pursue these matters, I will explain why R = ERR is compatible with RIC.

Note first that RIC is the contributory counterpart to *ought implies can* (OIC). According to OIC, if a person ought to do something, she can do that thing. Since ‘ought’ is a requiring notion, I take ‘reason’ as it figures in RIC to concern requiring reasons, rather than merely justifying reasons.

According to the proponent of R = ERR, that Nishi will be there is a (requiring) reason for Miyuki to go to the pub only if there is a fact which makes it wrong in some way for her not to go to the pub *and* that Nishi will be there is evidence of that fact. But, if Miyuki cannot go to the pub, it is not in any way wrong for her not to go. So, given E = ERR, that Nishi will be there is not a reason for Miyuki to go the pub.

The principle in play here is *wrong in a respect not to act implies can act*, which I take to be as plausible as RIC. From this principle, plus R = ERR, it follows that there is a (requiring) reason for a person to act only if she can act.^24^

## Problems of reasons as evidence

I have argued that R = ERR is able to avoid a number of objections which appear to undermine R = EO. There are, however, further challenges which any account of reasons as evidence faces. In this section, I will explore some of those challenges and suggest that they are unsuccessful or, at least, far from decisive.

### Enablers

A further objection to R = EO is that it collapses the distinction between reasons and *enabling conditions* (Brunero 2009; Brunero Forthcoming; Fletcher 2013).^25^ An enabler is not a reason for φing but a condition which, when it obtains, allows some other consideration to provide a reason for φing. For example, that Miyuki can go to the cinema is not a reason for her to go but it enables the fact that *Paris, Texas* is showing to provide such a reason. Were Miyuki unable to go to the cinema, that *Paris, Texas* is showing would not be a reason for her to go.^26^

The problem for R = EO is that there are circumstances in which the fact that Miyuki can go to the cinema is evidence that she ought to go. Suppose that, whenever Miyuki is able to go to the cinema, she ought to do so. Against that background, that she can go is evidence that she ought to go. R = EO predicts that it is a reason for her to do so; however, it is supposed to be an enabler in this context, not a reason. More generally, R = EO predicts that enablers are reasons in many cases.

Does R = ERR face a version of this problem? In some circumstances, that Miyuki can go to the cinema is not evidence of any respect in which it is right for her to go. So, according to R = ERR, it is not a reason for her to go in those circumstances. However, suppose that, whenever Miyuki is able to go the cinema, *Paris, Texas* is showing. Against this background, that Miyuki can go to the cinema is evidence that it will bring her pleasure. Hence, it is evidence of a respect in which it is right for Miyuki to go. Hence, given R = ERR, it is a reason for her to go.

In view of this, it is not clear that R = ERR has an advantage over R = EO when it comes to addressing the issue at hand. By way of response, I will make two points.

First, distinguishing reasons from enablers seems to be a problem for everyone. Consider the view that reasons are right-makers. If it is right for Miyuki to go to the cinema, that she can go contributes to making this the case.^27^ Consider next the view that reasons are premises of good reasoning. That she can go could be a premise in Miyuki’s reasoning as to whether to do so.^28^ Finally, consider the view that reasons are primitive. When the fact that Miyuki can go is evidence that her favourite film is showing, it seems to cast going to the cinema in a favourable light. It is unclear on what grounds the primitivist can deny that it is a reason.

To be clear, these remarks are not intended as serious challenges to the views I mention, and, of course, the survey is not exhaustive. The point is just that the difficulty of distinguishing reasons from enablers looks like a general one, and not particular to any account of reasons.

Be that as it may, there is a case for thinking that, if R = ERR predicts that in some cases enabling conditions are reasons, that is a welcome result. As all parties agree, if there is one thing a reason does, it is to speak in favour of acting. And the fact that Miyuki can go to the cinema, when it is evidence that *Paris, Texas* is showing, casts going in a favourable light. So, the verdict that, when they are evidence of respects in which acting is right, enablers are reasons seems in accordance with our initial or pre-reflective grasp on the notion of a reason.

On any view of reasons, one and the same fact can play the role of both a reason for acting and a reason against acting. Consider again the fact that it is sunny, which is a reason to go outside and a reason not to do so. It is therefore unsurprising that one and the same fact can play the role of both an enabling condition and a reason.

### Testimony

Another objection to R = EO appeals to cases of testimony. Suppose that Andrea tells Todd that he ought to clap. If Andrea is a reliable judge of such things, her testimony is evidence that Todd ought to clap. But, the objection continues, it is not a reason for him to clap. So, it is false that, if a fact is evidence that a person ought to φ, it is a reason for her to φ.^29^

R = ERR faces a similar challenge. After all, in suitable circumstances, that Andrea tells Todd that he ought to clap might be evidence of a respect in which it is right that he claps, say, that it keeps a promise. Given R = ERR, it is a reason to clap. But, one might think, this is the wrong verdict.

While this objection receives the most attention in the literature concerning R = EO, it is far from decisive. Many, including philosophers who seek neither to advance nor to defend R = EO, simply do not share the intuition that testimony cannot provide a reason for action [see, for example, Lord (2017) and Markovits (2010)]. So, there is deadlock over this issue.

To make progress, it might help to ask what thought lies behind the objection or drives the relevant intuitions. The standard answer is that reasons are right-makers.^30^ If it is right for Todd to clap his hands, it is not because of Andrea’s testimony.

I agree that Andrea’s testimony does not make it right for Todd to clap his hands. But it is question-begging in the present context to appeal to the idea that reasons are right-makers in objecting to R = ERR. That is simply to oppose one account of reasons with another, namely, R = MR, an account, moreover, which I criticise above.^31^

### Evidence as normative

Some maintain that evidence is in some sense normative (cf. Kim 1988). That on its own need not be a problem for R = ERR or, for that matter, R = EO. It still might succeed in providing an account of reasons in terms of two other normative notions, rightness and evidence. However, some maintain that the concept of evidence *just is* the concept of a reason for belief. In that case, R = ERR is circular; it appeals to the very concept it seeks to explain. In the first instance, this is an objection to R = ERR as a conceptual thesis, although it might extend to R = ERR as a metaphysical thesis, given the assumption that, if the concept of evidence is normative, the property of being evidence is normative.

If the concept of evidence is the concept of being a reason for believing, R = ERR still succeeds in offering an account of *practical* reasons by appeal to *epistemic* reasons. Setting this aside, not all circularity is vicious. Even if evidence is to be understood in terms of reasons, the account might be illuminating or informative insofar as it traces significant connections among the relevant concepts, connections which are not otherwise apparent, or insofar as it has nontrivial consequences.

All of that said, I do not accept that the concept of evidence is normative in the relevant sense. Settling this issue is beyond the scope of this paper but I will offer some preliminary remarks. Kelly, an influential proponent of the view, writes:

On any view according to which evidence is a normative concept, there is no gap between possessing evidence that some proposition is true and possessing reasons to think that that proposition is true; in offering evidence for a conclusion one is ipso facto providing reasons to think that that proposition is true. In contrast, on the view of evidence [as non-normative], statements such as the following should have no whiff of paradox:

- I have overwhelming evidence that p is true. But I have no reason to think that p is true. (2007: 468)

These remarks are unpersuasive. First, if one denies that evidence is a normative concept, one can still agree that a person providing evidence for a proposition thereby provides a reason for believing it (that there is no ‘gap’ between the two). According to R = ERR, evidence for a proposition is a reason for believing it because it is evidence of a respect in which so believing is right (cf. fn14 above). Here, rightness is the normative notion, not evidence.

Second, suppose that it is absurd or confused to judge: I have overwhelming evidence that p but no reason for believing that p. A proponent of R = ERR can explain this without appeal to the idea that evidence is normative. This judgement reveals a failure to grasp the concept of a reason, not of evidence.

Third, it is not obviously confused or absurd to judge: I have overwhelming evidence that p but no reason for believing that p. Suppose that the person who judges this is a pragmatist and maintains that it is right to believe a proposition just in case doing so promotes wellbeing. On this view, evidence of the truth of a proposition is not evidence of a respect in which believing it is right. One might think that the pragmatist is mistaken but one need not think that she fails to grasp the concept of evidence, or that she employs it a way which fails to accord with its content. The pragmatist’s position is a substantive, first-order view, not a meta-normative one. It is comparable to the position of a hedonist who thinks that it is right to perform some act just in case doing so is pleasurable.^32^

In summary, if the concept of evidence is normative, the account of reasons I advance here might be circular, but that need not be a problem. However, the concept evidence is not normative, at least, not for the reasons Kelly gives.^33^

## Conclusion

In view of difficulties facing the idea that reasons are right-makers, I suggested that a reason for acting is evidence of a respect in which acting is right. This account differs from the view that a reason for acting is evidence that a person ought to act in two ways. First, it appeals to a contributory notion, not an overall notion. Second, it holds that the relevant evidence is evidence for a non-normative proposition, not a normative proposition. As a result, my proposal avoids the more serious difficulties facing its predecessor while enjoying its advantages, chief among them that it respects the wide-ranging roles that reasons play in our ethical lives.

## Footnotes

1 Some maintain that the reasons for which a person acts, her so-called *motivating* reasons, are her attitudes. It follows from this that motivating reasons cannot be normative reasons. Accommodating a view of this sort—for example, by talking of a person acting on the basis of motivating reasons which correspond to normative reasons—would affect the presentation but not the substance of these remarks.

2 For views in this ballpark, see Alvarez (2010), Broome (2013), Chappell (2012), Hyman (2015), Markovits (2010), and Schroeter and Schroeter (2009).

3 For this view, see Thomson (2008), Kearns and Star (2008, 2009).

4 Alternatives include: reasons are premises of good reasoning (Hieronymi 2005; McHugh and Way 2016; Setiya 2014; Way 2017); reasons are good bases for acting (Gregory 2016); reasons are explanations of why it is good that a person acts (Finlay 2014; Raz 1999); reasons are explanations of why acting promotes a person’s desires (Schroeder 2007); and reasons are primitive (Parfit 2011; Scanlon 1998; Skorupski 2010).

5 For discussion, see Dorsey (2012), Vogelstein (2012), Sylvan (2015) and Whiting (2014).

6 For discussion, see Comesaña and McGrath (2014), Schroeder (2008) and Lord (2010). It is controversial whether this distinction cuts across that between objective and subjective reasons.

7 This idea is implicit in many discussions of normative reasons; it is explicit in Alvarez (2010), Chappell (2012) and Schroeter and Schroeter (2009). Hyman (2015) claims that a reason to act is an *explanation* of why it is right to act. Whether this is equivalent to R = MR depends on how ‘explanation’ is understood. According to Hyman, whether one fact explains another depends on ‘the presumed knowledge and intellectual ability of the audience’ (2015: 136). But whether one fact obtains because of another does not depend on such things. Broome (2013) claims, roughly, that a reason for a person to act is an explanation of why she *ought* to act. Whether this is equivalent to R = MR depends, again, on how ‘explanation’ is understood, and also on the relationship between what a person ought to and what it is right for her to do. On the first issue, Broome says at one point that to explain something is to make it the case (48). Elsewhere, he suggests that whether one thing explains another depends on background knowledge (51). On the second issue, Broome claims in passing that the difference between what it is right for a person to do and what she ought to do is terminological (70n27). Note that both Alvarez and Broome revise or supplement their accounts of reasons to accommodate the point I introduce at the start of §3, that is, to accommodate (outweighed) contributory reasons (cf. fn12 below).

8 This does not beg any questions in the debate between so-called objectivists and their prospectivist, subjectivist, or perspectivist opponents. That debate concerns the *deliberative* notion of rightness, that is, the notion which figures in the question that frames or guides deliberation, ‘What is the right thing for me to do?’ [For this way of characterising the debate, see Zimmerman (2006).] Participants in this debate can, and typically do, agree that there is a fact-relative notion of rightness; they disagree on the substantive issue of whether the deliberative notion of rightness is the fact-relative notion.

9 Thanks to an anonymous referee for encouraging me to address this point.

10 For this distinction, see Baier (1995: 76–78). Compare Dancy’s (2004) distinction between ‘peremptory’ and ‘enticing’ reasons, Scanlon’s (2014) distinction between ‘non-optional’ and ‘optional’ reasons, and Hurka and Shubert’s (2012) distinction between ‘prima facie duties’ and ‘prima facie permissions’. A related idea, due to Gert (2007), is that reasons have two dimensions of *strength*—justifying and requiring.

11 The difficulty of explaining the contributory in terms of the overall is a theme in Dancy (2004).

12 Alvarez makes the same suggestion in different terms: ‘A reason for φing makes φing pro tanto right’ (2010: 17). Of course, there are other contributory notions to reach for, for example, that of being good in some respect or to some degree. For criticism, see Brunero (2013) and Way (2013). Broome (2013: §4.3) tells a rather different story, which I will not explore here. For criticism of Broome’s account of contributory reasons, see Brunero (2013), Brunero (Forthcoming), Gregory (2016) and Kearns and Star (2008: 42–44).

13 Even if every respect in which it is right to act is fundamentally a respect in which acting causes pleasure, say, it remains the case that an action can be right in some respects, and wrong in others. One and the same act might in one way cause pleasure and in another way not do so, but cause pain instead.

14 The difficulty here for R = MRR is even more apparent when considering the epistemic domain. Arguably, there is only one respect in which it is right to believe a proposition, namely, that it is true (see Boghossian 2008; Fassio 2011; McHugh 2014; Shah and Velleman 2005; Wedgwood 2013; Whiting 2010; Whiting 2013). But it is clear that this is not the only consideration we treat as a reason for believing.

15 Compare an objection Kearns and Star make to Broome. On Broome’s account, they claim, reasons ‘practically important’ (2008: 41, also Star 2015: 18). Their complaint is that Broome fails to explain or reveal what makes reasons apt to play the role they play in our ethical lives. My complaint is that R = MRR predicts that many of the things that play that role are not reasons. In a different context, Kearns and Star argue that, if Broome only allows fundamental right-makers to count as reasons, his view predicts the existence of too few reasons (2008: 48–49). My point is that, even if a proponent of R = MRR allows that non-fundamental right-makers are reasons, she will end up with too few reasons. While these points differ in letter, they clearly share in spirit.

16 R = ERR entails that facts are evidence. In support of this view, see Littlejohn (2012) and Williamson (2000). I can allow that there are other legitimate non-factive conceptions of evidence which might serve different explanatory purposes.

17 Admittedly, it sounds a bit odd to say that a fact is evidence of itself. I doubt that this runs very deep. If one were to insist that that p is not evidence that p, one could revise the account of reasons to accommodate this: the fact that p is a reason for a person to φ if and only if it is, or is evidence of, a respect in which it is right for her to φ. For the view that a fact can be evidence for itself, see Williamson (2000: 187).

18 Kearns and Star present R = EO as a metaphysical thesis, while Thomson present it, in the first instance, as a conceptual thesis. Again, this does not make a great deal of difference in what follows.

19 There are other accounts of reasons in the same ballpark as R = ERR and R = EO, although the routes by which their proponents arrive at those accounts and their defences of them are in each case different. I will briefly mention those of which I am aware. Sharadin (2016) argues that a reason *of the right kind* for an attitude is evidence that it is correct to have that attitude. This differs from R = ERR in several respects. First, Sharadin’s is an account of reasons for attitudes, not actions. Second, it is not a general account of reasons but of reasons of the right kind. [For discussion of the distinction between the right and wrong kinds of reason, see Hieronymi (2005).] Third, Sharadin maintains, in keeping with R = EO but in contrast to R = ERR, that what makes something a reason is that it is evidence for a normative proposition, namely, that the relevant attitude is correct. Finally, Sharadin’s account, like R = EO and unlike R = ERR, appeals to an overall, as opposed to contributory, notion. Silverstein (2015) suggests that a reason for acting is evidence that the relevant action will maximize the aim of practical reasoning. My view makes no mention of reasoning, hence, of reasoning’s aim. Also, on Silverstein’s account, like R = EO and unlike R = ERR, what makes something a reason is that it is evidence for an overall verdict, namely, that the relevant act maximises the relevant aim. Finally, while R = EO corresponds to Thomson’s account of reasons for action, she offers a distinct account of reasons for attitudes. According to it, a reason for an attitude is evidence for a proposition, the truth of which makes that attitude correct (2008: ch. 8). This is close to R = ERR but, again, it is an account of reasons for attitudes, not actions. More importantly, Thomson’s proposal, unlike mine, involves an overall notion, that of correctness, rather than a contributory notion.

20 One issue is how to extend it to reasons which are evidence of respects in which it is right to φ but not evidence of respects in which it is wrong not to φ (that is, how to extend it to merely justifying reasons).

21 To say that, in this case, there is no reason for Miyuki to break her promise, despite the evidence for the anti-Rossian view, is not to take a stand in the recent debate about normative uncertainty [see, for example, Harman (2015) and Sepielli (2009)]. My claim is that, in the above case, there is no *objective* reason for Miyuki not to go to the pub, whereas the issue in the aforementioned debate concerns whether there is a *subjective* reason for Miyuki not to go.

22 Star (2015: 102–105) considers cases involving misleading normative evidence as posing a challenge to his theory of virtue, which is not my present concern. The discussion is brief and the focus lies elsewhere but Star seems to allow that, in such cases, the supposedly misleading evidence can give rise to genuine reasons for acting. This, it seems to me, is a cost for R = EO.

23 For defence of RIC, see Streumer (2007).

24 One might insist that *justifying reason implies can*. This principle is the contributory counterpart to *may implies can*, which lacks the pedigree of OIC and, in my view, is less plausible. Be that as it may, to accommodate the principle, a proponent of R = ERR can appeal to the idea that *right in a respect to act implies can act*. From this, plus R = ERR, it follows that there is a justifying reason for a person to act only if she can act.

25 Dancy (2004) stresses the importance of distinguishing reasons from enablers.

26 As noted above, Kearns and Star deny that *reasons implies can*; so, they presumably deny that the fact that a person can φ is an enabler for any reason there is for her to φ. However, the point requires only that there is a distinction between reasons and enablers; it does not depend on any particular example.

27 As Schroeter and Schroeter (2009: 286) note.

28 As Setiya (2014) notes.

29 For versions of this objection, see Broome (2008), Brunero (Forthcoming), McBride (2013), McKeever and Ridge (2012) and McNaughton and Rawling (2011). For Kearns and Star’s responses, see Kearns and Star (2008: 51, 2011, 2013a, b: 74–71).

30 For variations on this theme, see Broome (2008), Kearns and Star (2009: 233) and McNaughton and Rawling (2011).

31 For further criticism of R = MR, see Brunero (Forthcoming) and Brunero (2013).

32 On some views, like Shah’s (2006), it is part of the concept of belief that it is right (or correct) to believe a proposition if and only if it is true. This does not help the proponent of the view that evidence is a normative concept. According to Shah, the pragmatist is confused about the concept of *belief*, not the concept of evidence.

33 One might suspect that the account is circular in a different way, namely, that rightness is to be understood in terms of reasons. Again, if the account is circular, that need not be a problem. For arguments that rightness is primitive, see Chappell (2012) and McHugh and Way (2016). Note that R = EO is open to a similar objection, since it is plausible that what a person ought to do is understood in terms of reasons.
